# Gd-EOB-DTPA-enhanced MRI for evaluation of liver function: Comparison between signal-intensity-based indices and T1 relaxometry

**DOI:** 10.1038/srep43347

**Published:** 2017-03-07

**Authors:** Michael Haimerl, Niklas Verloh, Florian Zeman, Claudia Fellner, Dominik Nickel, Sven A. Lang, Andreas Teufel, Christian Stroszczynski, Philipp Wiggermann

**Affiliations:** 1 Department of Radiology, University Hospital Regensburg, Regensburg, Germany; 2 Center for Clinical Trials, University Hospital Regensburg, Regensburg, Germany; 3 MR Applications Predevelopment, Siemens AG, Healthcare GmbH, Erlangen, Germany; 4 Department of Surgery, University Hospital Regensburg, Regensburg, Germany; 5 Department of Internal Medicine I, University Hospital Regensburg, Regensburg, Germany

## Abstract

Gadolinium ethoxybenzyl-diethylenetriaminepentaacetic acid (Gd-EOB-DTPA) is a paramagnetic hepatobiliary magnetic resonance (MR) contrast agent. Due to its OATP1B1/B3-dependent hepatocyte-specific uptake and paramagnetic properties increasing evidence has emerged to suggest that Gd-EOB-DTPA-enhanced MRI can be potentially used for evaluation of liver function. In this paper we compare the diagnostic performance of Gd-EOB-DTPA-enhanced relaxometry-based and commonly used signal-intensity (SI)-based indices, including the hepatocellular uptake index (HUI) and SI-based indices corrected by spleen or muscle, for evaluation of liver function, determined using the Indocyanin green clearance (ICG) test. Simple linear regression model showed a significant correlation of the plasma disappearance rate of ICG (ICG-PDR) with all Gd-EOB-DTPA-enhanced MRI-based liver function indices with a significantly better correlation of relaxometry-based indices on ICG-PDR compared to SI-based indices. Among SI-based indices, HUI achieved best correlation on ICG-PDR and no significant difference of respective correlations on ICG-PDR could be shown. Assessment of liver volume and consecutive evaluation of multiple linear regression model revealed a stronger correlation of ICG-PDR with both (SI)-based and T1 relaxometry-based indices. Thus, liver function can be estimated quantitatively from Gd-EOB-DTPA–enhanced MRI-based indices. Here, indices derived from T1 relaxometry are superior to SI-based indices, and all indices benefit from taking into account respective liver volumes.

Quantitative evaluation of liver function in clinical routine is crucial for both monitoring patients with chronic liver disease and preoperative assessment of hepatic functional reserve in patients undergoing major liver resection. To date, the evaluation of biochemical blood parameters and clinical scoring systems like the Model for End-Stage Liver Disease (MELD) score or Child- Pugh Score as well as the preoperative liver volumetry is used for assessing liver function and for selecting appropriate patients for liver surgery^1^,^2^,^3^. In this context, considered to be the goldstandard for assessment of liver function, the indocyanine green (ICG) clearance test has been established as a valuable tool^4^. However, these methods do not consider the heterogeneous functional distribution of liver parenchyma and therefore only provide a global assessment of liver function. A tool that would allow for both providing anatomical information and estimating segmental liver function prior to surgery would be desirable.

Gadoxetic acid (Gd-EOB-DTPA; Primovist^®^, Bayer Healthcare, Berlin) is a paramagnetic hepatobiliary magnetic resonance (MR) contrast agent for T1-weighted imaging^5^. After iv injection, approximately 50% of the injected dose undergoes specific OATP1B1/B3- dependent hepatocyte uptake with consecutive biliary excretion via multidrug resistance protein 2 (MRP2) at the canalicular membrane of hepatocytes^6^. Here, using the OATP1B1/B3 – MRP2 pathway, Gd-EOB-DTPA and ICG are dependent on the same transport mechnisms; therefore, similar to ICG clearance, gadoxetate disodium-enhanced MR imaging should provide information for quantitative evaluation of liver function and allow for anatomic delineation of hepatic function^7^,^8^,^9^.

Several previous studies have shown that patients with impaired liver function or advanced liver fibrosis presented with decreased liver parenchymal enhancement and a relationship between various biochemical and clinical parameters indicating liver function and signal- intensity (SI)-based measurements after Gd-EOB-DTPA administration has been demonstrated. Here, for signal intensity measurements, the relative enhancement of the liver as well as various SI ratios, corrected by signal intensity of spleen or muscle have been used^10^,^11^,^12^,^13^,^14^,^15^,^16^,^17^. Yamada *et al*. recently reported that MRI-based estimation of liver function using liver parenchymal enhancement can be improved by taking into account the respective liver volumes and introduced the hepatocellular uptake index (HUI)^18^.

However, SI measurements provide relative parameters on an arbitrary scale which can neither be compared between repeated examinations of the same patient, nor between different patients. Moreover, SI measurements are depenedent on many technical parameters like the radiofrequency amplifier and the used receiver coils^19^. B1-field heterogeneity, repetition times (TR), and respiratory motion all influence the observed SI and the clinical applicability is challenging due to the nonlinear relationship between the gadolinium concentration and MR-signal intensity^20^,^21^.

Providing absolute, comparable parameters, the evaluation of T1 relaxation time is an alternative approach to the direct measurement of SI and has recently received augmented attention as a diagnostic tool for quantitative evaluation of liver function. Few studies have demonstrated that Gd-EOB-DTPA-enhanced MR imaging using T1 Relaxometry can be helpful for estimating liver function according to the MELD score or Child-Pugh score in patients with chronic liver disease or cirrhosis^12^,^19^,^22^,^23^. In this context, our study group recently has shown that liver function as determined using ICG-test can be estimated quantitatively using Gd-EOB-DTPA-enhanced T1 relaxation times and respective liver volumes^24^. However, in the current literature, discordant opinions exist regarding the most applicable parameter derived from Gd-EOB-DTPA-enhanced MRI for assessment of liver function.

The purpose of this study was therefore to compare the diagnostic performance of Gd-EOB-DTPA-enhanced T1 relaxometry and commonly used SI-based indices for evaluation of liver function, determined using the ICG test and Child-Pugh Score.

## Results

### Demographic and clinical data

**Figure 1:**
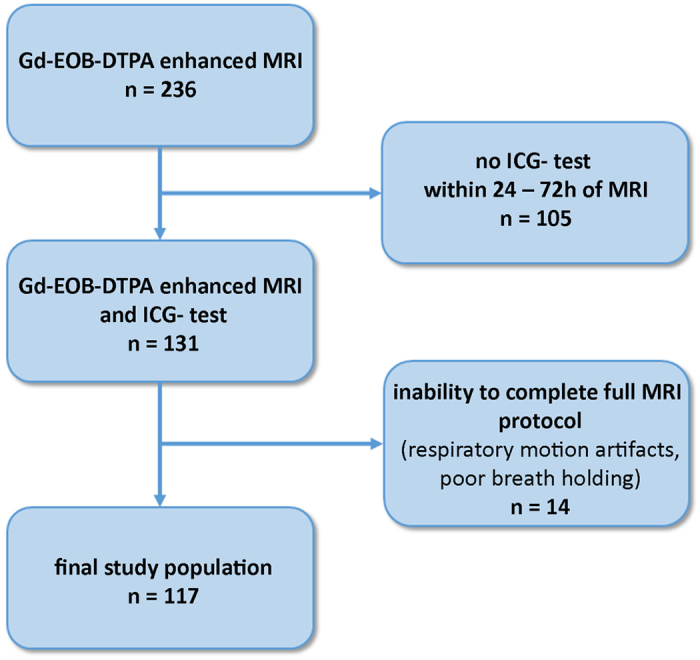
Flowchart of included patients.

Patient characteristics stratified by the Child-Pugh score are summarized in Table 1 and included study population is provided in Fig. 1.

Mean liver volume was significantly higher in normal liver patients and patients with Child Pugh A compared to patients with Child-Pugh B (p = 0.001; p = 0.003). Furthermore the mean age was significantly lower in case of NLF than in patients with Child Pugh A and B, respectively (p = 0.001; p = 0.004) (Table 2).

No significant difference was found for the other demographic variables between the patients with different Child Pugh scores.

### Child Pugh score

Stratified by the Child-Pugh Score, SI measurements as well as the reduction rate of T1 relaxation time (rrT1) and ΔR1 were lower for patients with reduced liver function showing a constant significant decrease with progression of liver cirrhosis (CPA, CPB, CPC). Patients with normal liver function as well as patients with increasingly impaired liver function were correlated with ICG-PDR (r = 0.89). With increasing progression of liver cirrhosis score, a significant decrease of ICG-PDR could be shown. Signal intensities and T1 values for all patients stratified by the Child-Pugh score are shown in Table 2.

### SI- based liver function indices

The predictive power of respective liver function indices on ICG-PDR was analyzed.

**Figure 2:**
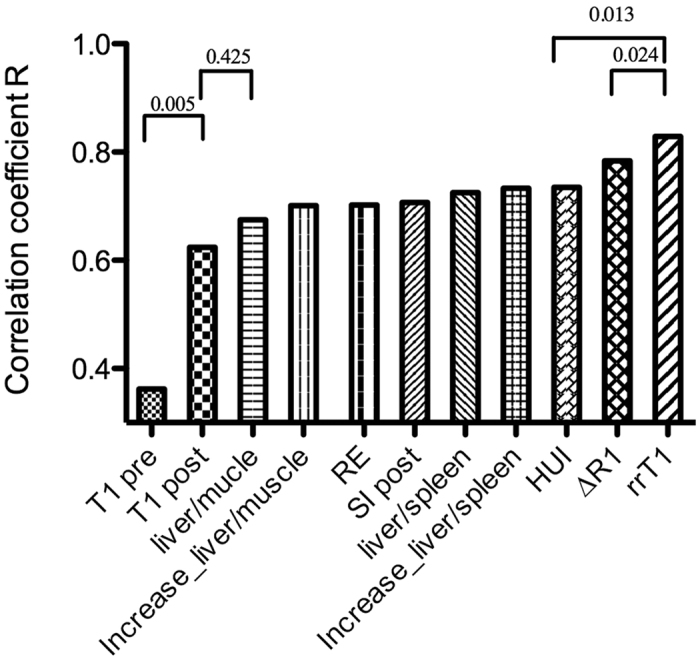
Comparison of correlation coefficients R between signal intensity- and relaxometry based indices. Correlation coefficients among log (ICG-PDR) and liver function indices have been calulated in a simple linear regression analysis. Reduction rate of T1 relaxation time (rrT1) shows significantly higher correlation coefficient (r = 0.83) compared to all signal intensity based indices (p ≤ 0.013). T1 pre, T1 relaxation time before Gd-EOB-DTPA administration; T1 post, T1 relaxation time after Gd-EOB-DTPA administration; liver/muscle, liver-to-muscle ratio; Increase_liver/muscle, increase rate of the liver-to-muscle ratio; RE, relative enhancement; SI post, signal intensity 20 min after Gd-EOB-DTPA administration; liver/spleen, liver-to-spleen ratio; inrease_liver/spleen, increase rate of the liver-to-spleen ratio; HUI, hepatocellular uptake index; ∆R1, inverse T1 relaxation time; rrT1, reduction rate of T1 relaxation time.

All Gd-EOB-DTPA-enhanced MRI-based liver function indices using signal intensities of liver before and 20 min after Gd-EOB-DTPA administration as well as SI-indices corrected by spleen or muscle correlated significantly with ICG-PDR (r = 0.675 to 0.733, p < 0.001; Fig. 2, Table 2). In a simple regression analysis the optimal curve fit for ICG-PDR was a log-linear relationship with best correlation of the increase rate of the liver-to-spleen ratio among SI-based liver function indices without inclusion of respective liver volume (LV) (r = 0.733) (Table 3).

Taking into account LV the hepatocellular uptake index (HUI) was calculated and multiple linear regression models for all SI-based liver function indices, including LV as an additional covariate, were constructed. Since LV is part of the HUI, it was included in the linear regression model. In this model, HUI correlated significantly with ICG-PDR (r = 0.735) (Table 3) and revealed no statistical significant better prediction for ICG-PDR compared to the increase rate of the liver-to-spleen ratio, even without consideration of liver volumes (r = 0.733, p = 0.953).

For multiple regression analysis, again, the optimal curve fit for ICG-PDR was a log-linear relationship and the best predictor among SI-based liver function indices was obtained by considering the liver-to-spleen ratio and respective liver volumes (r = 0.767) (Table 4).

**Figure 3:**
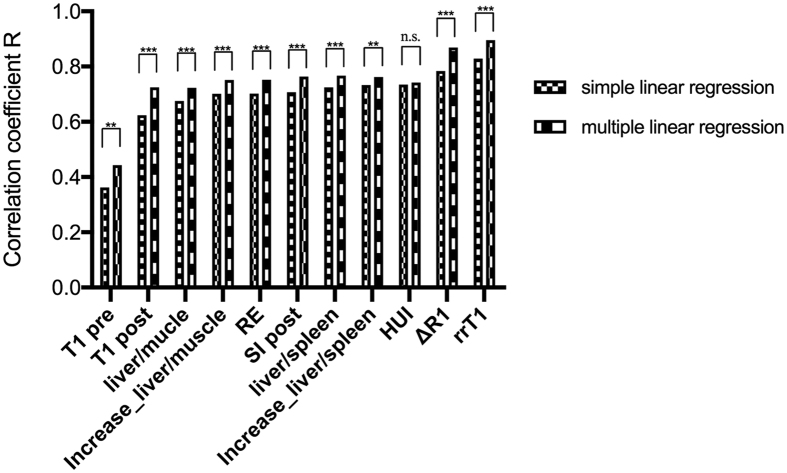
Comparison of correlation coefficients R of liver function indices and log (ICG-PDR) calculated in a simple linear regression model and a multiple regression model under the consideration of respective liver volumes, respectively. T1 pre, T1 relaxation time before Gd-EOB-DTPA administration; T1 post, T1 relaxation time after Gd-EOB-DTPA administration; liver/muscle, liver-to-muscle ratio; Increase_liver/muscle, increase rate of the liver-to-muscle ratio; RE, relative enhancement; SI post, signal intensity 20 min after Gd-EOB-DTPA administration; liver/spleen, liver-to-spleen ratio; inrease_liver/spleen, increase rate of the liver-to-spleen ratio; HUI, hepatocellular uptake index; ∆R1, inverse T1 relaxation time; rrT1, reduction rate of T1 relaxation time. ***p ≤ 0.001; **p ≤ 0.01; n.s. non significant.

All SI-based liver function indices revealed better correlation with ICG-PDR taking into account respective liver volumes in multiple regression analysis (without LV; r = 0.675 to 0.733, with LV; r = 0.723 to 0.767, Fig. 3).

### T1 relaxometry based liver function indices

The reduction rate of the T1 relaxation time (rrT1) of the liver correlated most strongly with the ICG-PDR among all Gd-EOB-DTPA-enhanced MRI-based liver function indices.

**Figure 4:**
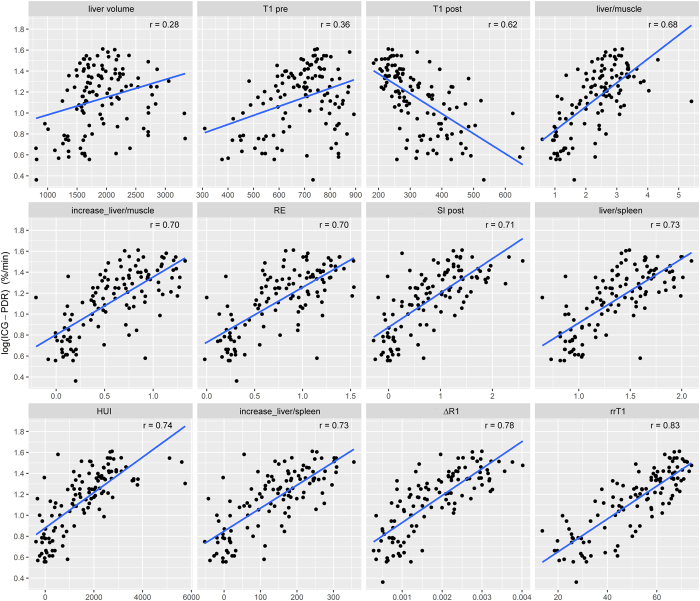
Correlation analysis of signal-intensity- and relaxometry-based liver function indices on ICG-PDR. Scatterplots of liver volume (r = 0.28), T1 relaxation time 20 min before Gd- EOB-DTPA (T1_pre,_ r = 0.36), T1 relaxation time after Gd-EOB-DTPA administration (T1 post, r = 0.62); the liver-to-muscle ratio (r = 0.68); the increase rate of the liver-to-muscle ratio (r = 0.70); the relative enhancement (RE, r = 0.70); the signal intensity 20 min after Gd-EOB-DTPA administration (SI post, 0.71); the liver-to-spleen ratio (r = 0.73); the hepatocellular uptake index (HUI, r = 0.74); the increase rate of the liver-to-spleen ratio (r = 0.73); ∆R1 (r = 0.78); and the reduction rate of T1 relaxation time (rrT1, r = 0.83) on log (ICG-PDR) (%/min).

The correlation of rrT1 and ΔR1 with ICG-PDR was investigated. The optimal curve fit for ICG-PDR was a log-linear relationship with r = 0.829 and 0.784, respectively (Fig. 4). A better prediction was obtained by considering the respective liver volumes and a multiple linear regression model was constructed in a second step. The r value changed from 0.829 to 0.90 (p < 0.001) for rrT1 and from 0.784 to 0.869 for ΔR1in the log (ICG-PDR) model (Fig. 3).

**Figure 5:**
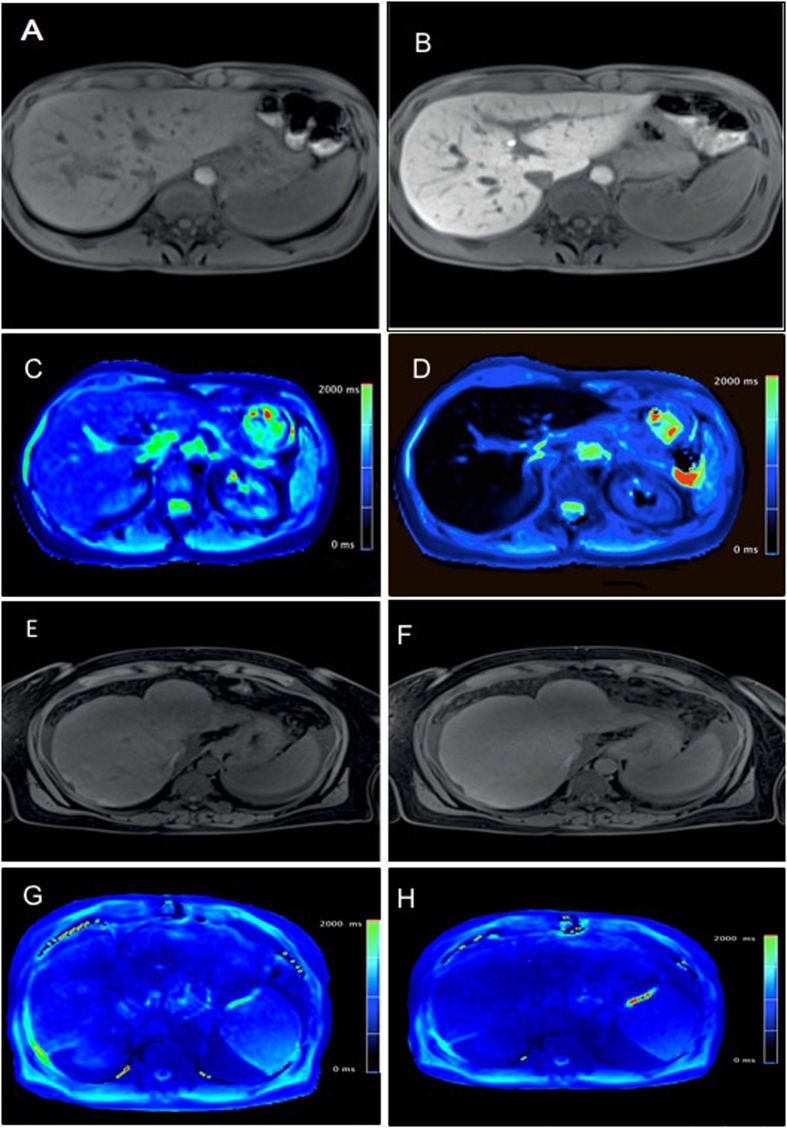
T1-weighted VIBE sequences and colour-coded T1 maps for patient with normal and impaired liver function. T1 weighted VIBE sequences (**A**,**B**,**E**,**F**) and T1 maps (**C**,**D**,**G**,**H**) with inline correction for B1 heterogeneities were obtained before and 20 min after the administration of Gd-EOB-DTPA in a 45-year-old patient with normal liver function (**A**–**D**; ICG-PDR = 29.1%/min) and in a 62-year-old patient with impaired liver function (**E**–**H**; ICG-PDR = 3.6%/min).

T1-weighted VIBE sequences and colour-coded T1 maps for patient with normal and impaired liver function are shown in Fig. 5.

## Discussion

Gd-EOB-DTPA-enhanced MRI is already used in an increasing number of patients scheduled for liver surgery allowing both delineation of tumors from liver parenchyma as well as assessment of topography with regards to intrahepatic vessels. In recent years, increasing evidence has emerged to suggest that Gd-EOB-DTPA- enhanced MRI can be potentially used for evaluation of global and remnant liver function^12^,^13^,^15^,^18^,^19^,^24^,^25^,^26^,^27^,^28^,^29^. The use of Gd-EOB-DTPA-enhanced MRI could therefore provide both morphological and potentially functional information of the liver in a single examination.

The indices that have been proposed for assessment of liver function using Gd-EOB-DTPA-enhanced MRI include both SI-based indices and T1 relaxometry. Here, SI-based indices are corrected by a variety of parameters to compensate the relative nature of an arbitrary unit, e.g. the SI before contrast media administration or the signal intensities of the spleen or muscle.

In a prospective study we recently could demonstrate that Gd-EOB-DTPA-enhanced T1 relaxometry in combination with respective liver volumes might be a valuable tool for estimating liver function as determined using ICG-test^24^.

However in the current literature, there still exists no consensus on which parameters derived from Gd-EOB-DTPA-enhanced MRI – either SI-based indices or T1 relaxometry-based indices- are the most suitable for evaluation of liver function. We therefore designed an retrospective study comparing all known MRI-based indices, which contains in parts data from our previously published study concerning T1 relaxometry values (overlap of patients: n = 174).

It is important to recognize that Gd-EOB-DTPA uptake does not correlate with various clinical factors (weight, height, gender, kidney function, bilirubin)^30^. Especially the repeated hyothesis that unconjugated bilirubin levels do compete in a marked way with Gd-EOB-DTPA uptake can not be supported: the well known organic anion- transporting ploypeptides OATP1B1 and OATP1B3 mediate the bulk of intracellular Gd-EOB-DTPA uptake. In direct comparison OATP1B3 has a more than 6 fold higher affinity towards Gd-EOB-DTPA as compared to OATP1B1, thus being the most important way of Gd-EOB-DTPA uptake into the hepatocyte. On the other hand unconjugated bilirubin is transported via the cell membrane of hepatocytes by various transporters (mostly OATP2) but not via OATP1B3 and thus is not competing markedly with Gd-EOB-DTPA uptake^8^,^31^,^32^.

In our study, all SI-based indices for the estimation of liver function (RE, liver-to-spleen ratio, liver-to-muscle ratio, increase rate of the liver-to-spleen ratio, increase rate of the liver-to-muscle ratio) significantly correlated with the ICG-PDR (p < 0.001; r = 0.675–0.733), with the weakest correlation for the liver-to-muscle ratio (r = 0.675) and the highest correlation for the increase rate of the liver-to-spleen ratio (r = 0.733) Compared to our results, Utsunomiya *et al*. reported a compareable correlatation between ICG-R15 and the liver-to-muscle ratio (r = 0.67)^29^, whereas the results of Kamimura *et al*. revealed a weaker correlation (r = 0.319)^10^.

Using precontrast image data, individual differences of signal intensities in the liver, muscle or spleen before Gd-EOB-DTPA administration may be reduced, which should allow for precise assessement of Gd-EOB-DTPA liver parenchyma uptake. Taking into account the SI of muscle before contrast media administration, the increase rate of the liver-to-muscle ratio (r = 0.701) correlated more strongly with ICG-PDR than the liver-to-muscle ratio alone (r = 0.675) which is consistent with the results of Kamimura *et al*.^10^, however, this difference was not statistically significant in our study (p = 0.506).

In the literature, few studies reported an increase rate of a liver-to-muscle ratio, also called “contrast enhancement index” (CEI)^17^ and corrected liver enhancement ratio^14^, respectively, on liver fibrosis showing divergent correlations with the stage of liver fibrosis according to the METAVIR score with correlation coefficients ranging from 0.322 to 0.79^14^,^16^,^17^, and Yoneyama *et al*. provided a correlation coefficient of 0.528, compared to ICG-R15^12^.

While our results revealed a good correlation between ICG- parameter and the liver-to-spleen ratio (r = 0.725), which is arithmetically equivalent to the corrected enhancement of the liver-to-spleen ratio provided by Yoneyama *et al*. (r = 0.457)^12^, both Motosugi *et al*. and Kamimura *et al*. showed weaker correlations (r = 0.428, r = 0.405)^10^,^11^.

Calculating an increase rate of the liver-to-spleen ratio taking into account the SI of spleen before contrast media administration, which is arithmetically equivalent to the liver-to-spleen-contrast ratios (LCS_N20) provided by Noren *et al*.^33^ and Yoneyama *et al*.^12^, our results revealed a slightly better correlation with ICG-PDR compared to the liver-to- spleen ratio alone; however, the difference was not statistically significant (r = 0.725 vs. r = 0.733, p = 0.799).

In this study, the correlation (r = 0.702, p < 0.001) between the RE of the liver and ICG parameter (ICG-PDR) was stronger compared to the correlation coefficients in the literature provided in previous reports by Yoneyama *et al*. (0.520; p < 0.001)^12^, and Kubota *et al*. (r = 0.462; p = 0.003)^34^. In this context, a few studies compared liver function as expressed by different clinical scoring systems (model for endstage liver disease (MELD) Score, Child- Pugh Score) with RE of the liver after Gd-EOB-DTPA administration and showed strong correlation between RE and liver function^13^,^14^,^15^,^35^.

However, MRI SI measurements reflect relative values on an arbitrary scale and depend on many technical parameters, such as the used pulse sequence, reconstruction method or MRI system manufacturer, the potency of the radiofrequency amplifiers, and the used receiver coils^19^. SI measurements can neither be compared between different patients nor between repeated examinations of the same patient, and may vary considerably at each imaging time point. Moreover, it has been shown, that the relationship between gadolinium concentration and MR signal intensities is not linear^21^. Conversely, measurement values derived from T1 relaxometry are not affected by these different factors providing absolute, comparable values (unit: ms).

Indeed, among all provided Gd-EOB-DTPA-enhanced MRI-based liver function indices, our results revealed that the reduction rate of the T1 relaxation time (rrT1) of the liver correlated most strongly with ICG-PDR (r = 0.829). The correlation was significantly higher compared to the increase rate of the liver-to-spleen ratio, which has been shown to provide the best correlation coefficient among the SI values without liver volumes (increase rate of liver-to-spleen ratio, r = 0.733, p < 0.014).

Previous studies showed that measuring the LV is a useful tool for estimating liver function and that a decrease of the liver volume comes along with the progression of liver cirrhosis^36^,^37^. Moreover, Hashimoto and Watanabe *et al*. showed a proportional relationship between ICG-parameters and the total hepatic parenchymal cell volume^38^. Because the LV is dependent on both the total hepatic parenchymal cell volume, depending on the patient’s physical constitution, and the progression of chronic liver disease, a correction of the MRI-based liver function indices for the volume of the liver should improve the prediction of liver function, expressed by ICG parameters.

In our study, the LV showed a weak correlation with the ICG-PDR (r = 0.283, p = 0.002).

In a multivariate analysis, we correlated all SI-based indices and values derived from T1 relaxometry in combination with the respective liver volumes with ICG-parameters and observed significantly higher correlations for all indices compared to those without the inclusion of respective liver volume (p ≤ 0.003).

Yoneyama *et al*. correlated ICG-R15 with various MRI-based liver function indices using SI measurements or T1 relaxation time; however, they did not provide higher correlations for the liver-to-spleen ratio (without LV: r = 0.525; with LV: r = 0.498) and the reduction rate of T1 relaxation time (without LV: r = 0.574; with LV: r = 0.559) by taking into account respective liver volumes^12^. One possible reason might be the simple multiplication of liver function indices with respective liver volumes without using the information a multivariate analysis model does provide. This might underestimate the added value of the liver volume.

Taking into account respective liver volumes for quantitative evaluation of liver function, Yamada *et al*. introduced the hepatocellular uptake index (HUI), a corrected enhancement of the liver-to-spleen ratio multiplied by the liver volume (V_L_ × (L20/S20) − 1). Here, the contrast enhancement effect of Gd-EOB-DTPA caused by the extracellular fluid space, which is the sum of the intravascular and extravascular spaces, is corrected by including the SI of the spleen^18^. However, the authors did neither correlate the ICG-PDR with the liver-to-spleen ratio by itself nor with the relative enhancement. Thus, the added value of the liver volume remains ambiguous. Moreover, Yamada *et al*. assumed a linear relationship between HUI and ICG-PDR providing a correlation coefficient of r = 0.87. However, contrary to the retention rate of ICG after 15 min (ICG-R15; unit: %), the diffusion rate of ICG (ICG- PDR) is subject to the influence of the measured timespan and the respective unit is provided in %/min. Our results revealed that the correlation between ICG-PDR and liver function indices, either SI-based or derived from T1 relaxometry, follows an exponential distribution with consecutive log-linear relationship. Therefore, Yamada *et al*. might have underestimated the correlation between HUI and ICG-PDR.

In this study, among indices without involvement of respective LV, the reduction rate of T1 relaxation time showed stronger correlation with ICG-PDR (r = 0.829) compared to HUI (r = 0.735) with statistically significant difference between the correlations of rrT1 and HUI (p = 0.013). Using respective liver volumes in a multivariate analysis model, compared to HUI, all MRI-based liver function indices correlated stronger with ICG-PDR with highest values for the reduction rate of T1 relaxation time (r = 0.90), allowing for the calculation of an estimated ICG-PDR value.

Our study has several limitations. First, the number of included patients was small. A stronger correlation between Gd-EOB-DTPA-enhanced MRI-based liver function indices and ICG-PDR could have been obtained if our study had included a larger number of patients. Second, only one experienced radiologist performed ROI placement and ROI placement may be a source of variation due to the potential inhomogeneous distribution of parenchymal changes. However, averaging four repetitive ROI measurements across an area of the liver parenchyma should yield representative values. Moreover, to obtain homogeneous T1 maps of the entire liver, we used a prototype three-dimensional gradient echo triple-flip angle technique with the preceding acquisition of a B1 map for inline correction of B1 inhomogeneities. Yoneyama *et al*. used a three-dimensional dual-flip technique to acquire T1 maps in a spoiled gradient echo sequence lacking a B1 correction to counteract B1 heterogeneities. These reasons might contribute to the small, not significant differences between SI-based measurements and relaxometry measurements. Moreover, no histological evidence of hepatitis or liver fibrosis for most of the patients was available in our study.

In conclusion, liver function correlating with ICG-PDR can be estimated quantitatively using Gd-EOB-DTPA-enhanced MRI-based indices. Here, indices derived from T1 relaxometry are superior to SI-based indices, and all indices benefit from taking into account respective liver volumes. Our results indicate that Gd-EOB-DTPA-enhanced T1 relaxometry, in combination with liver volume, may have the potential to become a novel tool for monitoring liver function.

## Material and Methods

### Patients

Written informed consent was obtained from all participating patients and the study was performed in accordance with the relevant guidelines and regulations. The local institutional review board approval of the University Hospital Regensburg was obtained for this retrospective study.

The current retrospective analysis is based in parts on a previously published patient cohort (overlap: 174 patients, new patients: 62)^24^.

The aim and scope of the current study differs markedly compared to the previous study insofar as we have a different context of data analysis. Whilst our previously published study was a prospective study to establish a new liver function parameter based on T1 relaxometry, the current study is a retrospective comprehensive comparison and evaluation of all published MRI- based liver function tests. Thus parts of the following Materials and Methods section have a close resemblance to our previously published study, with regards to the definition of basic study parameters, i.e. imaging sequences, ICG-test, the definition of the Child Pugh Score.

Retrospective analysis covered the period of time between end of August 2013 and April 2014 and included all patients who underwent both an ICG test for evaluation of liver function (n = 131) and a Gd-EOB-DTPA-enhanced MRI at 3.0 T (n = 236). To be included in the study, patients had to have undergone a Gd-EOB-DTPA-enhanced MR imaging examination with prototype T1 relaxometry sequences using variable flip angles that was performed within 24–72 h of ICG test.

14 patients were excluded from this study due to the inability to complete the full MR imaging protocol due to poor breath-holding and due to images with respiratory motion artifacts or severe imaging artifacts that would not allow analysis in T1 relaxometry images (Fig. 1).

All patients had no history of previous reaction to liver-specific MRI contrast media or contraindication to both MRI (e.g., claustrophobia, pacemaker) and Gd-EOB-DTPA administration in terms of no renal failure (defined as a glomerular filtration rate of less than 30 mL/min), respectively. None of the patients had a thyroid hyperfunction or an iodine allergy.

Finally, 117 patients (79 men and 38 women; mean age, 55.5 ± 13.5 years) were included in our study. 50 patients had a normal liver function (NLF) with ICG-PDR > 16.0%/min and no evidence or clinical signs of liver cirrhosis. 67 patients were assigned to the liver cirrhosis group, where diagnosis of liver cirrhosis was based on laboratory values, imaging findings and clinical findings according to the Child-Pugh Score depending on the degree of prothrombin time (INR, 1 point, <1.7; 2 points, 1.71–2.3; 3 points, >2.3), serum albumin (g/dl, 1 point, >3.5; 2 points, 2.8–3.5; 3 points, <2.8), serum bilirubin (mg/dl, 1 point, <2; 2 points, 2–3; 3 points, >3), ascites (1 point, none; 2 points, mild; 3 points, moderate to severe) and encephalopathy (1 point, none; 2 points, Grade I–II; 3 points, Grade III–IV). 27 of the 67 patients with liver cirrhosis were classified with Child-Pugh A (CPA, 5–6 points), 22 patients with Child-Pugh B (CPB, 7–9 points), and 18 patients with Child-Pugh C (CPC, 10–15 points). Patient characteristics are shown in Table 1.

### MR imaging

All imaging was performed using a clinical whole body 3T system (Magnetom Skyra, Siemens Healthcare, Erlangen; Germany) and combination of spine and body array coils (18-channel body matrix coil, 32-channel spine matrix coil) was used for signal reception. Each T1-weighted volume-interpolated breath-hold examination (VIBE) sequence with fat suppression (repetition time (TR), 3.09 ms; echo time (TE), 1.16 ms; flip angle, 9°; parallel imaging factor, 2; slices, 64; reconstructed voxel size, 1.3 × 1.3 × 3.0 mm; measured voxel size, 1.7 × 1.3 × 4.5 mm; acquisition time, 14 s) was acquired during breath-hold before and 20 min after Gd-EOB-DTPA (Primovist; Bayer Healthcare, Berlin, Germany) administration. All patients received a Gd-EOB-DTPA dose (0.025 mmol/kg body weight) adapted to their body weight, which was administered via bolus injection with a flow rate of 1 mL/s and flushed with 20 mL NaCl. Every sequence covered the entire liver before Gd-EOB-DTPA administration and in hepatobiliary phase after 20 min (HBP).

In addition to the routine imaging protocol, T1 mapping of the liver was performed before Gd-EOB-DTPA administration and in HBP using a prototypical VIBE sequence (TR 5.79 ms, TE 2.46 ms) using variable flip angles (1°, 7°, 14°) and a voxel size of 3.6 mm × 2.5 mm × 4.7 mm, interpolated to 2.5 mm × 2.5 mm × 3.0 mm. A B1 map of the whole liver was acquired for each patient before T1 mapping to improve the homogeneity of the T1 maps, and color-coded T1 maps were calculated inline. The whole liver was covered in 17 s during breath-hold using an acceleration factor of 4 and CAIPIRINHA (Controlled Aliasing In Parallel Imaging Results in Higher Acceleration) as a parallel imaging technique.

### Image analysis

The mean SI values on T1-weighted images of the liver, spleen and paravertebral muscle (at the hight of the liver hilum) and T1 relaxation time on T1 maps of the liver, obtained before and 20 min after Gd-EOB-DTPA administration, were measured by using operator-defined region-of-interest (ROI). ROIs were manually placed at identical locations in every sequence, excluding liver lesions, visible blood vessels, or imaging artifacts. Four ROIs (3 in right lobe, 1 in left lobe) were located in VIBE images and T1 maps and one ROI was located in the paravertebral muscle and spleen, before and after administration of Gd-EOB-DTPA, respectively. The mean signal intensities and T1 values for the four ROIs in the liver were regarded as the representative SI and T1 values of the entire liver, respectively. Each ROI was a circle that was chosen as large as possible and ROIs were manually adjusted between sequences (before and after Gd-EOB-DTPA administration) in the case of patient movement. The size of the ROIs ranged from 0.9 to 4.2 cm^2^ in liver parenchyma, 0.8–2.1 cm^2^ in paravertebral muscle and 2.7–4.9 cm^2^ in spleen. To avoid measurement bias, the radiologist was blinded to the clinical, haematological and other radiological information. From the data acquired, the following Gd-EOB-DTPA-enhanced MRI-based liver function indices using SI or T1 relaxation time before and 20 min after Gd-EOB-DTPA administration were calculated:

1. Hepatocellular uptake index (HUI)^18^, which is:
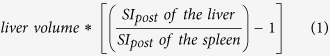
2. Relative enhancement of the liver^15^,^34^, which is:
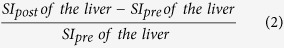
3. Liver-to-spleen ratio^10^,^11^,^14^, which is:
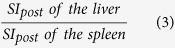
4. Liver-to-muscle ratio^10^,^29^, which is:
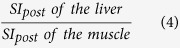
5. Increase rate of the liver-to-spleen ratio^16^, which is:
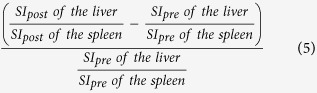
6. Increase rate of the liver-to-muscle ratio^16^,^10^, which is:
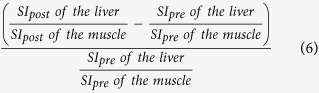
7. Reduction rate of T1 relaxation time of the liver^19^,^24^, which is:
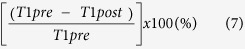
8. ΔR1 of the liver^28^, which is:

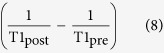


Considering the volumes of respective livers, volume-assisted indices of signal intensity and T1 relaxometry could be calculated using a multivariate regression analysis.

### Volumetric analysis

To draw the outlines of the liver on every section, the delayed T1- weighted 3D VIBE sequence hepatobiliary phase was used. In this procedure, MR images were provided on an Aquarius iNtuition Viewer (TeraRecon Inc., San Mateo, CA, USA) on a commercially available workstation and free-hand contours were used to obtain respective liver volumes. If there was a liver tumour that was hypointense on 20-min-delayed Gd-EOB-DTPA-enhanced images, the volume of the hypointense tumour was excluded from the volume of the liver parenchyma.

### ICG Test

To determine the ICG plasma disappearance rate (PDR) the noninvasive pulse-densitometric LiMON system (Impulse Medical System, Munich, Germany) was used. A bolus dose of 0.5 mg per kilogram bodyweight of ICG (ICGPulsion, Munich, Germany) was injected into a cubital vein, followed by 10 mL of normal saline flushed through the intravenous catheter, and each patient was monitored with an ICG finger clip connected to the liver function monitor (LiMON) via an optical probe. Injected ICG was detected from fractional pulsatile changes in optical absorption, and ICG elimination measurements (ICG-PDR) were determined by monoexponential transformation of the original ICG concentration curve and backward extrapolation to time point zero (100%), describing the decay as a percentage change with time.

To acquire liver function within a reasonable timeframe and to eliminate any confounding with Gd-EOB-DTPA enhanced MRI, the ICG-test of patients was performed 24–72 h prior to or 24–72 h post-MRI.

### Statistical analysis

A one-way analysis of variance (ANOVA) was used to analyze differences in continuous variables between patients with NLF and patients with different stages of liver cirrhosis. Post hoc pair-wise comparisons were made with the Tukey procedure. Simple and multiple linear regression models were calculated to determine the predictive power of Gd-EOB-DTPA-enhanced signal intensity indices and T1-relaxometry measurements on the ICG-parameter ICG-PDR. Since ICG-PDR is an exponential transformation of the ICG-R15, which is linear related to the SI- and T1-relaxometry based liver function indices, the logarithm of ICG-PDR was used as dependent variable in all models. The optimal curve fit was assessed visually and using the coefficient of correlation r. Correlation coefficients were compared using the method proposed by Steiger. The statistical significance level was set to 0.05 (two-sided). All statistical analyses were carried out using IBM SPSS Statistics (version 23, Chicago, IL, USA) and R 3.0.3.

## Additional Information

**How to cite this article:** Haimerl, M. *et al*. Gd-EOB-DTPA-enhanced MRI for evaluation of liver function: Comparison between signal-intensity-based indices and T1 relaxometry. *Sci. Rep.* **7**, 43347; doi: 10.1038/srep43347 (2017).

**Publisher's note:** Springer Nature remains neutral with regard to jurisdictional claims in published maps and institutional affiliations.

## Figures and Tables

**Table 1:** Patient characteristics.

Patients	All (n = 117)	NLF (n = 50)	CPA (n = 27)	CPB (n = 22)	CPC (n = 18)
Age (years)	55.1 ± 13.5	50.6 ± 14.0	62.0 ± 11.3	59.5 ± 7.9	54.5 ± 16.4
Gender					
Male	79 (67%)	25 (50%)	21 (78%)	19 (86%)	14 (78%)
Female	38 (33%)	25 (50%)	6 (22%)	3 (14%)	4 (22%)
Height (m)	1.72 ± 0.08	1.72 ± 0.08	1.71 ± 0.08	1.73 ± 0.08	1.73 ± 0.08
BMI (kg/m^2^)	26.5 ± 4.9	26.8 ± 5.1	26.8 ± 3.8	26.8 ± 4.4	25.0 ± 4.4

NLF: normal liver function; CPA: Child-Pugh A; CPB: Child-Pugh B; CPC: Child-Pugh C.

**Table 2:** Signal intensities and T1 relaxometry values.

Patients	All (n = 117)	NLF (n = 50)	CPA (n = 27)	CPB (n = 22)	CPC (n = 18)
Mean liver volume (ml)	1896.2 ± 507.3	1999 ± 380	1922 ± 457	1609 ± 477	1922 ± 775
SI liver pre	171.8 ± 32.2	180.7 ± 32.5	183.3 ± 27.8	160.0 ± 238.0	144.3 ± 26.0
SI liver post	307.1 ± 98.6	366.4 ± 77.3	335.8 ± 70.8	238.0 ± 70.7	184.1 ± 37.5
SI muscle pre	126.0 ± 27.6	119.0 ± 24.0	121.7 ± 27.7	137.8 ± 28.0	137.6 ± 29.4
SI muscle post	139.5 ± 30.0	130.0 ± 25.4	136.8 ± 31.3	153.8 ± 31.1	152.5 ± 29.1
SI spleen pre	134.3 ± 24.9	130.2 ± 23.9	142.6 ± 26.1	136.6 ± 22.2	130.6 ± 27.7
SI spleen post	175.6 ± 33.2	163.2 ± 30.6	188.7 ± 36.1	189.5 ± 30.9	174.0 ± 25.3
T1 pre (ms)	681.2 ± 126.4	712.5 ± 77.6	720.8 ± 105.4	646.7 ± 162.5	576.8 ± 153.4
T1 post (ms)	326.1 ± 98.9	261.3 ± 57.4	341.6 ± 82.5	368.9 ± 63.4	430.7 ± 123.2
Mean ICG-PDR (%/min)	16.7 ± 9.8	25.9 ± 6.6	14.6 ± 4.1	7.7 ± 2.9	5.6 ± 2.6

NLF: normal liver function; CPA: Child-Pugh A; CPB: Child-Pugh B; CPC: Child-Pugh C.

**Table 3:** Simple linear regression model of MRI-based indices on ICG-PDR.

	Simple linear regression model of MRI-based indices on ICG-PDR		
	B (95%-CI)	p-value	r
Signal-intensity based indices			
SI post	0.002 (0.002–0.003)	<0.001	0.707
RE	0.524 (0.426–0.623)	<0.001	0.702
Liver/muscle	0.228 (0.182–0.274)	<0.001	0.675
Liver/spleen	0.327 (0.269–0.385)	<0.001	0.725
Increase_liver/muscle	0.550 (0.447–0.654)	<0.001	0.701
Increase_liver/spleen	0.613 (0.507–0.719)	<0.001	0.733
HUI	0.017 (0.014–0.020)	<0.001	0.735
T1 relaxometry based indices			
T1 pre	0.086 (0.045–0.127)	<0.001	0.362
T1 post	−0.190 (−0.234–0.146)	<0.001	0.624
ΔR1	257.8 (220.15–295.47)	<0.001	0.784
rrT1	0.016 (0.014–0.018)	<0.001	0.829

**Table 4:** Multiple linear regression model of MRI-based indices and liver volume on ICG-PDR.

	B (95%-CI)	p-value	r
SI post	0.002 (0.002–0.003)	<0.001	0.764
LV	0.017 (0.010–0.024)	<0.001	
RE	0.520 (0.429–0.612)	<0.001	0.752
LV	0.016 (0.009–0.023)	<0.001	
Liver/muscle	0.225 (0.181–0.268)	<0.001	0.723
LV	0.015 (0.008–0.023)	<0.001	
Liver/spleen	0.327 (0.272–0.381)	<0.001	0.767
LV	0.015 (0.008–0.022)	<0.001	
Increase_liver/muscle	0.546 (0.450–0.643)	<0.001	0.751
LV	0.016 (0.009–0.023)	<0.001	
Increase_liver/spleen	0.602 (0.501–0.704)	<0.001	0.762
LV	0.012 (0.005–0.020)	<0.001	
T1 pre	0.082 (0.042–0.121)	<0.001	0.443
LV	0.015 (0.005–0.025)	<0.001	
T1 post	−0.205 (−0.245–(−0.166))	<0.001	0.725
LV	0.022 (0.014–0.030)	<0.001	
ΔR1	271.7 (241.4–302.1)	<0.001	0.869
LV	0.022 (0.017–0.028)	<0.001	
rrT1	0.016 (0.015–0.018)	<0.001	0.895
LV	0.020 (0.015–0.025)	<0.001	
